# The emblematic South African therocephalian *Euchambersia* in China: a new link in the dispersal of late Permian vertebrates across Pangea

**DOI:** 10.1098/rsbl.2022.0222

**Published:** 2022-07-13

**Authors:** Jun Liu, Fernando Abdala

**Affiliations:** ^1^ Key Laboratory of Vertebrate Evolution and Human Origins of Chinese Academy of Sciences, Institute of Vertebrate Paleontology and Paleoanthropology, Chinese Academy of Sciences, Beijing 100044, People's Republic of China; ^2^ College of Earth and Planetary Sciences, University of Chinese Academy of Sciences, Beijing 100049, People's Republic of China; ^3^ Unidad Ejecutora Lillo, Conicet-Fundación Miguel Lillo, Miguel Lillo 251, Tucumán, Argentina; ^4^ Evolutionary Studies Institute, University of the Witwatersrand, Johannesburg 2050, South Africa

**Keywords:** Therocephalia, Lopingian, Naobaougou Formation, China and South Africa

## Abstract

Therapsids were widely distributed in Pangea in the late Permian. South Africa in Gondwana and Russia in Laurasia are the principal areas recording tetrapods (including therapsids) of this age. More recent field explorations have increased the importance of Chinese late Permian fossil assemblages. This is clearly reflected in the discovery of several new therocephalians from the Naobaogou Formation in Nei Mongol. Here, we report a therocephalian from that unit identified as a new species of the emblematic South African taxon *Euchambersia*. The new species, *Euchambersia liuyudongi*, is represented by a well-preserved skull and mandible showing a well-developed maxillary fossa and the absence of postcanine teeth. This is the third akidnognathid therocephalian recovered from the Naobaougou Formation, but oddly, the two basal Chinese akidnognathids previously known were recovered from a younger unit of the formation than the derived *E. liuyudongi*. This is the first time that the same therocephalian genus has been recorded in northern and southern continents, making the record of the Naobaougou Formation key to understanding the evolution of late Permian continental fauna in general, and of akidnognathid therocephalians in particular.

## 1. Introduction

The transition from the Palaeozoic to Mesozoic is one of the most important periods in earth life history, as evidenced by the end-Permian mass extinction process, the most devastating phenomenon of this kind [1,2]. Non-mammaliaform therapsids were a crucial group of amniotes well represented at that time, and although they experienced a great loss of diversity, they continued to be well represented at the beginning of the Mesozoic, particularly during the Triassic [3,4].

Here, we document the occurrence of one of the most distinctive therapsids in the Lopingian of China. The closest relative of this new Chinese taxon, *Euchambersia mirabilis*, a putative venomous non-mammaliaform therapsid, is known from two partial skulls from the *Cistecephalus* Assemblage Zone (approx. 257 Ma) of the South African Karoo Basin [5–8]. Key features of *Euchambersia* are the presence of an enormous maxillary fossa deeply carving the surface of the maxilla behind the canine and the absence of postcanine dentition. The discovery of this taxon in China significantly enhances knowledge of *Euchambersia* as the new material is represented by a well-preserved complete skull and mandibles. This record is also another clear indication of the wide distribution of Lopingian therapsid taxa in Pangean faunas.

Institutional abbreviations: BP, Evolutionary Studies Institute, University of the Witwatersrand, Johannesburg, South Africa; IVPP, Institute of Vertebrate Paleontology and Paleoanthropology, Chinese Academy of Sciences, Beijing, China; NHMUK, Natural History Museum, London, UK.

## 2. Systematic palaeontology

Therapsida Broom 1905

Therocephalia Broom 1903

Eutherocephalia Hopson & Barghusen 1986

Akidnognathidae Nopcsa 1928

*Euchambersia* Broom 1931

### (a) Revised diagnosis

Autapomorphies within Therocephalia: broad excavation in the maxilla immediately posterior to the canine; pterygoid transverse flange reduced, lacking suborbital vacuity; the absence of upper and lower postcanines (also in *Theriognathus*).

**Figure 1.**
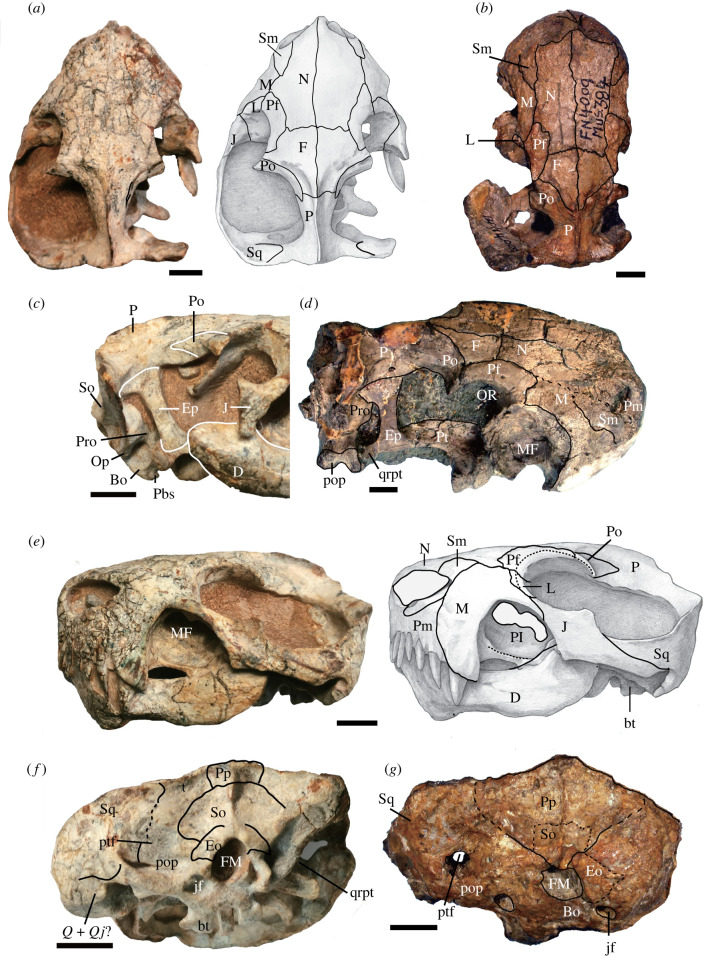
*Euchambersia* skull. *Euchambersia liuyudongi* holotype, IVPP V 31137, in dorsal view (*a*), right temporal region in lateral view (*c*), left lateral view (*e*) and occipital view (*f*); *Euchambersia mirabilis*, BP/1/4009, in dorsal view (*b*), and occipital view (*g*); holotype of *Euchambersia mirabilis*, NHMUK PV R5696, in dorsolateral view (*d*). Abbreviations: Bo, basioccipital; bt, basal tubera; D, dentary; Eo, exoccipital; Ep, epipterygoid; FM, foramen magnum; J, jugal; jf, jugular foramen; L, lacrimal; M, maxilla; MF, maxillary fossa; N, nasal; Op, opisthotic; OR, orbit; Pl, palatine; Pm, premaxilla; Pbs, parabasisphenoid; Po, postorbital; pop, paraoccipital process; Pp, postparietal; Pf, prefrontal; Pro, prootic; ptf, post-temporal foramen; Pt, pterygoid; *Q* + *Qj*, quadrate + quadratojugal; qrpt, quadrate ramus of the pterygoid; Sm, septomaxilla; So, supraoccipital; Sq, squamosal. Scale bar equals 10 mm.

*Euchambersia liuyudongi* n. sp. (figure 1*a*,*c*,*e*,*f*).

### (b) Etymology

Species name dedicated to Mr Liu Yu-Dong, the technician who discovered the specimen.

### (c) Holotype

IVPP V 31137, a nearly complete skull with lower jaw and a few postcranial bones (six vertebrae and a few rib fragments).

### (d) Type locality and age

Member I of Naobaogou Formation, Inner Mongolia (field locality 2020DQS-1); Wuchiapingian, Lopingian, Permian.

### (e) Diagnosis

Different from *E. mirabilis* in the shorter snout, prefrontal separated from the postorbital;­ maxillary fossa connected to nasal cavity; epipterygoid separated from prootic; post-temporal fenestra slit-like.

## 3. Description

The Chinese skull measures 70 mm in dorsal length, slightly shorter than BP/1/4009 from South Africa [6]. It has a complete left zygomatic arch and a relatively wide temporal fenestra. This fenestra is longer than that of BP/1/4009, and clearly shorter than that of NHMUK PV R5696 (figure 1*a*,*b*,*d*). The snout is short, less than 40% of the skull length, whereas it extends for more than half of the skull length in NHMUK PV R5696 and BP/1/4009 (figure 1*a*,*b*,*d*).

The tall premaxilla has a narrow exposure on the lateral side (figure 1*e*) and as in *E. mirabilis*, bears five narrow, spatulated upper incisors, which are slightly curved lingually, with conspicuous distal ridges.

The large external nares are close-set and face anteriorly. The large septomaxilla is well exposed on the dorsal surface with a well-developed posterodorsal facial process interposed between nasal and maxilla (figure 1*a*,*e*).

In lateral view, the maxilla has a tall facial plate. Its ventral margin forms a concave step between the canine and last incisor, with the base of the former placed much more ventrally than the base of the incisors (figure 1*e*). This difference in placement of incisors and canine bases is also present in NHMK PV R5696. The canine is conical and slightly larger than the fifth incisor, with no evidence of a ridge or groove. The lateral surface of the maxilla is deeply excavated posterior to the canine to form an oval maxillary fossa that reaches almost the mid-height of the snout (figure 1*e*). There are no upper postcanine teeth. Dorsally, the maxilla–nasal suture is relatively short, and the maxilla makes a tiny contact with the prefrontal (figure 1*a*). Posteriorly, a long triangular maxillary process, below the very short lacrimal, contacts the anterior process of the jugal (figure 1*e*). This portion is dorsoventrally low but mediolaterally wide, forming the suborbital bar and dorsally capping the maxillary fossa. The fossa is not exposed in dorsal view (figure 1*a*). The lateral margin of the maxillary fossa is formed mainly by the maxilla and the jugal, and the medial wall of the fossa is perforated by a large fenestra that communicates with the nasal cavity (figure 1*e*).

The dorsal surface of the skull is mainly composed of the paired nasals, frontals, and a single parietal featuring a sharp parietal crest that lacks a pineal foramen (figure 1*a*). Of these bones, the nasals are the longest, extending for half of the skull length, and the parietal is the shortest.

The long prefrontal in *E. liuyudongi* is separated from the postorbital by the frontal, different from *E. mirabilis* (figure 1*a*,*b*). The postorbital forms a short supraorbital rim and a long posterior projection on top of the frontal in the anterior third of the temporal fenestra (figure 1*a*).

The jugal is a slab-like bone with triangular dorsal (postorbital) and ventral (suborbital) processes (figure 1*e*). Posteriorly, it extends below the anterior process of the squamosal near to the posterior margin of the skull.

The right epipterygoid is well exposed above the right quadrate process of the pterygoid. The topmost dorsal end is strongly expanded, particularly along its posterior margin (figure 1*c*), whereas most of its dorsoventral extension, including its ventral end is only slightly expanded. Its posterior margin has no apophysis for contacting the prootic (figure 1*c*) and its posterodorsal margin is also separated from the anterodorsal margin of the prootic.

The lambdoidal crest is nearly straight, and the highest parts are close to the midline. As in *E. mirabilis*, a mid-crest lies above the large foramen magnum, extending over the supraoccipital and the postparietal (figure 1*f*,*g*). The post-temporal fenestra is slit-like, different from the rounded one of *E. mirabilis* (figure 1*g*).

Both dentaries are preserved, with the right more complete, but postdentary bones are lost (figure 1*c*,*e*). The dentary is bowed, with a high horizontal ramus and a low coronoid process terminating below the orbit (figure 1*c*). The lateral surface of the dentary is smooth and strongly convex. There are no lower postcanine teeth.

## 4. Discussion

The presence of a large excavation in the maxilla (maxillary fossa) immediately posterior to the canine in the Chinese specimen is a key character producing a very particular condition of the snout, previously only known in the South African therocephalian *E. mirabilis* [6]. *Euchambersia liuyudongi* has a short snout compared to *E. mirabilis* (figure 1). The ratio of the temporal fenestra length to the basal skull length is similar in the larger specimen of *E. mirabilis* (NHMUK PV R5696), but much larger than the similar-sized BP/1/4009. The temporal fenestra is relatively large in larger specimens of *E. mirabilis*, a trend also detected in the large sample of *Theriognathus* [9]. As in *E*. *mirabilis* and also *Theriognathus*, the new species does not feature upper postcanines and the finding for the first time of a mandible for *Euchambersia* confirms the expected absence of lower postcanine teeth.

There are a number of distinguishing features that suggest that the *Euchambersia* from China is a new species: the prefrontal is separated from the postorbital by the frontal,­ deeply excavated maxillary fossa such that the medial wall is perforated connecting with the nasal cavity, the maxillary fossa is not visible in dorsal view and the epipterygoid does not contact the prootic.

The most remarkable feature characterizing *Euchambersia* is the large maxillary fossa in the snout behind the canine, which historically was interpreted as lodging a venomous gland [5,8]. Recent detailed morphological studies of the two known specimens of the taxa using computed tomography (CT) and a wide exploration of functionality [6], concluded that a venomous gland was the most plausible hypothesis, although not without some difficulties. For us the major problem, also discussed by Benoit *et al*. [6], is the absence of a venomous gland similarly located (in the snout, in front of the orbit) in any other tetrapod and the existence of preorbital (scent) glands lodged in comparable structures (although clearly not sculpted as deeply on the snout) of some artiodactyls [6]. A first glimpse of the structure of the dentition of *E. liuyudongi* does not reveal in any element externally features that can be interpreted as being used for the delivery of poison. With this information, we are inclined at this time, to support the scent gland hypothesis. We will expand this work in the future by producing CT scans that will add to the knowledge of the dentition and the skull of this new species.

## 5. Phylogeny of Therocephalia

A parsimony analysis using the TNT program [10] produced 612 most parsimonious trees (mpt) of 462 steps. The strict consensus shows a large polytomy at the base of Therocephalia, and nearly all the lineages are in some way recovered (e.g. Scylacosauridae, Baurioidea, part of Akidnognathidae) except for Whaitsioidea (see electronic supplementary material, figure S1). The new Chinese taxon forms a monophyletic group with *E. mirabilis* of South Africa as a deeply nested member of akidnognathids. The majority consensus tree produces an extra level of resolution, with *Lycosuchus* recovered as the most basal therocephalian followed by scylacosaurids, and then *Gorynychus* placed as a stem eutherocephalian (a condition that previous phylogenies bestowed on *Scylacosuchus*). Eutherocephalia are represented by a lineage formed by Perplexisauridae and Akidnognathidae, both recovered as monophyletic; and a clade including a polytomy represented by (i) *Scylacosuchus*, (ii) *Nanictidops* (*Purlovia*, *Caodeyao*) and (iii) Whaitsioidea and Baurioidea, both recovered as monophyletic (electronic supplementary material, figure S2). Support is poor for most major lineages except for Baurioidea and the more nested portion of Akidnognathidae (the portion of this group recovered as monophyletic in the strict consensus), both having a Bremer support of three.

**Figure 2.**
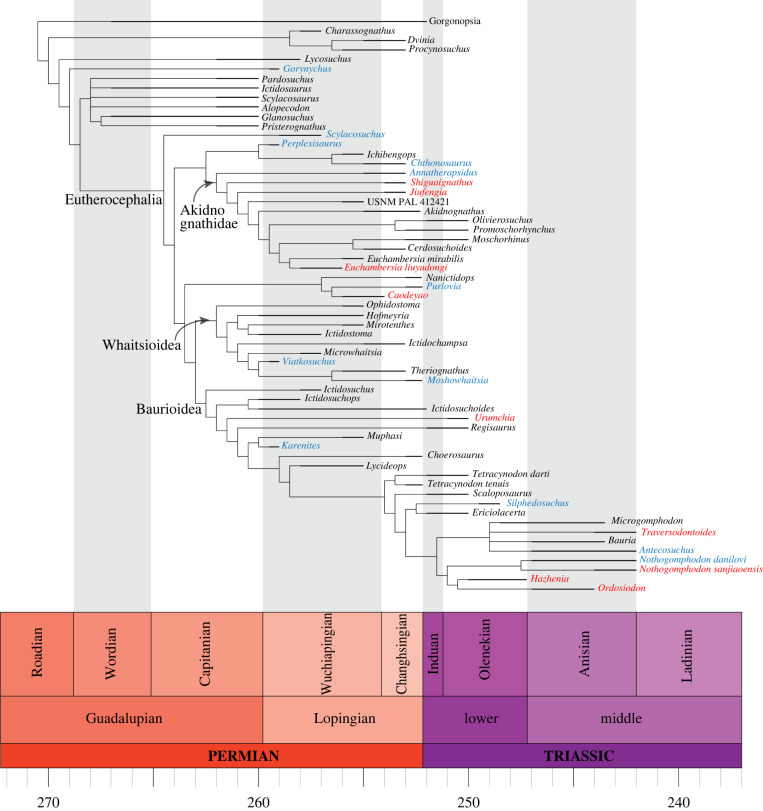
Calibrated strict consensus tree of Therocephalia using implied weight. Taxa from China in red, taxa from Russia in blue. Main therocephalian lineages indicated.

A second analysis using implied weight with concavity coefficient (*k*) set to 12 produced some interesting changes in the results. The analysis produced 24 mpt (fit 17.647). Strict and majority consensus show practically the same resolution (figure 2). *Lycosuchus* is recovered as the most basal therocephalian, followed by *Gorynychus*. The other difference regarding the previous analysis is that *Scylacosuchus* is here recovered as stem to Eutherocephalia, in accordance with most previous analyses. Finally, all the major lineages of Eutherocephalia are recovered as monophyletic (figure 2).

## 6. Conclusion

Sustained discoveries of late Permian therocephalians from China in the last 6 years [11–14] are providing a remarkably enhanced framework of knowledge of the group, as has been the case for Zambian therocephalians [15,16] and some new species from South Africa [17] and Russia [18] (electronic supplementary material, figure S4).

The most basal therocephalians recovered in our phylogeny are the South African *Lycosuchus* followed by the Russian *Gorynychus*. This placement of the Russian taxon contrasts with that presented by Kammerer & Masyutin [18], where *Gorynychus* was recovered as a stem eutherocephalian. This new placement suggests that the record of therocephalians in Laurasia started very early in the history of the group, a scenario supported by the presence of the fragmentary therocephalian *Porosteognathus efremovi* in the upper Wordian–Capitanian of Russia [19].

In all recent phylogenies [11,14,15,18], a basal clade, Scylacosauridae, has a distribution restricted to the Wordian–Capitanian of Gondwana (electronic supplementary material, figure S4). This represents the only therocephalian radiation limited to the Karoo Basin of South Africa. The same is true for middle Permian pareiasaurians, which are also limited to the Wordian–Capitanian of the Karoo Basin [20,21].

Most phylogenies recover the Russian *Scylacosuchus* from the early Wuchiapingian as a stem eutherocephalian. This particular age is pivotal in the evolution of the major lineages of the group. The majority of recent Chinese therocephalian finds are members of Akidnognathidae and the discovery of *E. liuyudongi* increases complexity in the history of the group. The current phylogeny reflects three taxa from the Northern Hemisphere as basal members of Akidnognathidae, while *E. liuyudongi* is nested in a more derived group that includes six South African taxa. Thus, the phylogeny is revealing the existence of another component of the Akidnognathidae history in China which, for now, is only represented by *E. liuyudongi*. The akidnognathid record of China is also distinct in that all the species discovered from the group are from the Naobaougou Formation and thus two different evolutionary stages of the Akidnognathidae are represented in the same unit. The record of *Euchambersia* in China, besides being the first genus of therocephalian shared between northern and southern Pangea faunas, is also the first deeply nested Akidnognathidae in north Pangea. Curiously, the two basal forms, *Jiufengia* and *Shiguaignathus*, were discovered in member III of the Naobaougou Formation [13,14], whereas *E. liuyudongi* from member I of the same formation is older.

Desert-like conditions existed in central Pangea during the Lopingian that did not completely interrupt tetrapod exchanges between the northern and southern regions of the world [22–24]. Following detailed taxonomic revisions (e.g. [25]), it is becoming increasingly clear that tetrapod late Permian distribution of genera from the northern and southern continents is rare, and reflected thus far in therapsids: dicynodonts *Diictodon, Lystrosaurus*, *Geikia* and the cynodont *Procynosuchus* (table 1). Four other cases of therapsid genera (three dicynodonts and one biarmosuchid) from southern Pangea were recovered in recent phylogenies as closely related to northern Pangea representatives [28–32]. Recently discovered new therocephalian genera in Zambia were also found to be closely related to Russian taxa, increasing the evidence of a close link between northern and southern faunas [15,16]. *Euchambersia* described here is the first evidence of a wide distribution of therocephalians in China and South Africa, reinforcing the idea of a sustained global distribution of therapsids, and illustrating that disparate, quite unusual morphotypes were represented on both sides of the world.

**Table 1.** Lopingian therapsids with genera shared between northern and southern Pangea. When different genera are listed they are recovered in phylogenies as deeply nested sister taxa.

Pangea north				Pangea south			references
China	Russia	Germany	Scotland	Tanzania	Zambia	South Africa	
*Diictodon*					*Diictodon*		[26,27]
*Daqingshanodon*						*Keyseria*	[28,29]
*Turfanodon*						*Dinanomodon*	[30,31]
	*Australobarbarus*					*Tropidostoma*	[31]
			*Geikia*	*Geikia*			[31]
	*Niuksenitia*					*Burnetia*	[32]
*Euchambersia*						*Euchambersia*	This paper
	*Lystrosaurus*					*Lystrosaurus*	[33–36]
	*Chthonosaurus*				*Ichibengops*		[15]
	*Karenites*				*Mupashi*		[16]
	*Moschowhaitsia*			*Theriognathus*			[9,37]
	*Procynosuchus*			*Procynosuchus*			[38,39]

Lopingian therocephalians from northern continents integrate all the major lineages of therocephalian known, but they are best represented by Akidnognathidae. The diversity of therocephalians at this time is clearly best expressed in the Southern Hemisphere in contrast with the diversity of the group in the Anisian which is greatest in the north of Pangea (figure 2). The fossil record of Lopingian therocephalians in the Northern Hemisphere of Pangea has seen significant changes in the past decade, with recent findings accentuating the contrast with non-mammaliaform cynodonts that continue to be poorly represented both in the late Permian and Triassic of the Northern Hemisphere.

## Data Availability

The data are provided in the electronic supplementary material [40].
